# Axonal Spectrins: Nanoscale Organization, Functional Domains and Spectrinopathies

**DOI:** 10.3389/fncel.2019.00234

**Published:** 2019-05-28

**Authors:** Cheng-Hsin Liu, Matthew Neil Rasband

**Affiliations:** ^1^Program in Developmental Biology, Baylor College of Medicine, Houston, TX, United States; ^2^Department of Neuroscience, Baylor College of Medicine, Houston, TX, United States

**Keywords:** spectrin, super-resolution microscopy, axonal excitable domains, axon integrity, spectrinopathy

## Abstract

Spectrin cytoskeletons are found in all metazoan cells, and their physical interactions between actin and ankyrins establish a meshwork that provides cellular structural integrity. With advanced super-resolution microscopy, the intricate spatial organization and associated functional properties of these cytoskeletons can now be analyzed with unprecedented clarity. Long neuronal processes like peripheral sensory and motor axons may be subject to intense mechanical forces including bending, stretching, and torsion. The spectrin-based cytoskeleton is essential to protect axons against these mechanical stresses. Additionally, spectrins are critical for the assembly and maintenance of axonal excitable domains including the axon initial segment and the nodes of Ranvier (NoR). These sites facilitate rapid and efficient action potential initiation and propagation in the nervous system. Recent studies revealed that pathogenic spectrin variants and diseases that protealyze and breakdown spectrins are associated with congenital neurological disorders and nervous system injury. Here, we review recent studies of spectrins in the nervous system and focus on their functions in axonal health and disease.

## Overview of Spectrins in the Nervous System

Spectrins were first isolated from the membranes of red blood cell ghosts (Marchesi and Steers, 1968) – in fact, the name spectrin derives from specter. Spectrins were identified as cytoskeletal proteins that confer elasticity to erythrocytes allowing them to withstand the shear forces experienced in the circulatory system (Elgsaeter et al., 1986). Since then, the functions of spectrins in diverse cell types were expanded to include signaling transduction (Hund et al., 2010), intracellular trafficking (Ikeda et al., 2006), and cellular polarity (Galiano et al., 2012).

Spectrins are expressed in all metazoan cells, but complicated tissues may express and localize spectrins in cell and compartment-specific manners. The human genome encodes two α-spectrins (*SPTA*, *SPTAN1*; αI and αII spectrin, respectively) and five β-spectrins (*SPTB, SPTBN1, SPTBN2, SPTBN4, SPTBN5*; βI-βV spectrin, respectively). In the nervous system, αII-spectrin (non-erythrocytic α-spectrin) is the sole α-subunit as verified at mRNA transcript (Zhang et al., 2014) and protein levels (Huang et al., 2017a). βI-spectrin is concentrated in cortical layer 2 and 4, cerebellar granule cells, and in the soma of Purkinje cells (Stankewich et al., 2010), while βII-spectrin is widely expressed in neurons and glia (Galiano et al., 2012; Zhang et al., 2013; Susuki et al., 2018). βIII-spectrin is found in the soma and dendrites of the cerebellar molecular layer (Stankewich et al., 2010). βIV-spectrin is concentrated at axon initial segments (AIS) and nodes of Ranvier (NoR) (Berghs et al., 2000). βV-spectrin is mainly expressed in the hair cell (Legendre et al., 2008) and photoreceptor (Papal et al., 2013). In this review, we will describe how new super-resolution microscopy techniques have facilitated a new appreciation for the function of spectrin cytoskeletons, what spectrins’ functions are in the axon during development, and how dysfunction of spectrins lead to neurological disorders.

## Structure and Domains of Spectrins

α-spectrins consist of a tetramerization motif at the N-terminus, followed by 21 tandem spectrin-repeats (SR), a Src homology domain 3 (SH3), a calmodulin-binding domain (only in αII-spectrin), and a calcium-binding EF-domain at the C-terminus. β spectrins consist of two actin-binding calponin homology (CH) domains (CH1-2) at the N-terminus, followed by 17 tandem SR [except for βV-spectrin, which contains 30 SR (Stabach and Morrow, 2000)], and a lipid-binding pleckstrin homology (PH) domain at the C-terminus. To mediate their interaction between membrane proteins and the actin-based cytoskeleton, SR 15 of β-spectrins binds to the ZU5 domains of ankyrins (Kennedy et al., 1991). βIV-spectrin also binds to a non-canonical phosphorylation-dependent site in ankyrinG’s giant exon (Jenkins et al., 2015). A pair of α/β-spectrins bind side-by-side to form a heterodimer. This is mediated by the SR 20-21 in α-spectrin and SR 1-2 in β-spectrin, although one recent study revealed a non-canonical dimerization domain in SR14-15 of βIV-spectrin (Huang et al., 2017a). Heterodimers further assemble head-to-head as a functional heterotetramer complex; this interaction is mediated by the N-terminus of α-spectrin and SR 17 of β-spectrin (Elgsaeter et al., 1986). In *Drosophila* and *Caenorhabditis elegans*, there is only one α subunit (Dubreuil et al., 1989) and two β subunits (β and β_H_-spectrin in *Drosophila*; β_G_ and β_H_-spectrin in *C. elegans*) (Dubreuil et al., 1987, 1990; McKeown et al., 1998; Moorthy et al., 2000). These α and β-spectrins are similar to their vertebrate homologs in structure and biophysical properties (Dubreuil et al., 1989; Moorthy et al., 2000).

## Visualizing the Spectrin Cytoskeleton by Super-Resolution Microscopy

The dynamic rearrangement of the cytoskeleton accommodates morphological changes and intracellular networks necessary to maintain cellular homeostasis. Highly polarized cell types like neurons undergo extensive re-organizations of cytoskeletal architecture to differentiate into cells with distinct functional and structural compartments such as axons and dendrites. Understanding how spectrins function together with other cytoskeletal components at the single molecule level has shed light on the detailed mechanisms of neuronal development, structure, and function.

Visualizing spectrin-dependent cytoskeletons at resolutions below that achieved by conventional light microscopy was first achieved using electron microscopy (EM) of the erythrocyte membrane (Byers and Branton, 1985). The approximately 200 nm rod structure of spectrin tetramers were crosslinked with junctional complexes to form hexagonal meshworks. Since then, EM-based approaches have been performed in cultured neurons (Jones et al., 2014) and tissues including outer hair cells (Legendre et al., 2008) and brain (Stankewich et al., 2010; Efimova et al., 2017). These studies provide valuable information about subcellular localization of spectrins with high-resolution images. However, the dense and compact nature of the cytoskeletal network limits clear description of the intricate architecture of neuronal spectrins. Furthermore, the special fixation procedures for sample preparation are relatively destructive, which makes it challenging to preserve the molecular integrity. Immunofluorescence microscopy-based assays can also uncover the spatial features of spectrins and their associated proteins in a variety of cellular compartments by combining multichannel chromophores. However, the resolution using immunofluorescence microscopy is limited to the optical diffraction limit of about 250 nm. Over the last decade, these limitations were gradually overcome through the use of advanced super resolution microscopy.

Super resolution microscopy can be categorized into three major types including: single-molecule-localization microscopy (such as stochastic optical reconstruction microscopy (STORM) and photoactivated localization microscopy (PALM)), periodic light pattern based structured illumination microscopy (SIM), and scanning technique based stimulated emission depletion microscopy (STED) (Sydor et al., 2015). Depending on the type of microscopy, the resolution can be anywhere from 10 to 130 nm in the xy plane, and 300 nm in the z-direction. Furthermore, by labeling molecules with photo-switchable fluorescent probes, accurate three-dimensional information of distinct molecules can be analyzed in multicolor images (Bates et al., 2007). Some imaging modalities also allow for live imaging and the temporal resolution can range from milliseconds to minutes, which confers the ability to visualize molecular dynamics. These strengths facilitate discoveries down to single molecule and supramolecular characterizations.

**FIGURE 1:**
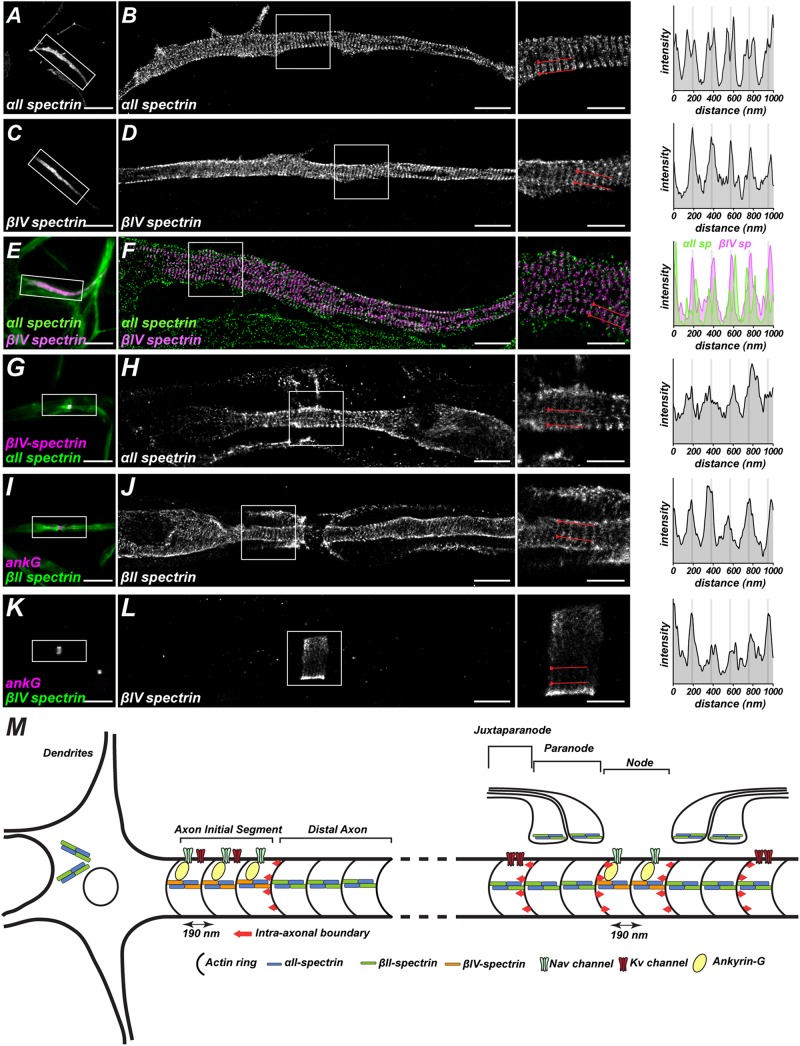
Spatial arrangements of spectrin-based cytoskeletons in the axon. **(A,C,E)** Images captured by conventional fluorescence microscopy show αII and βIV-spectrin at the axon initial segments of cultured hippocampal neurons. **(B,D,F)** Images captured by STORM super-resolution microscopy show a periodic lattice of αII and βIV-spectrin with spacing around 190 nm. The two-color DNA-PAINT images in **(F)** show αII-spectrin immunoreactivity flanks βIV-spectrin labeling, confirming that spectrins are arranged head-to-head in the spectrin tetramer. **(G,I,K)** Images captured by conventional fluorescent microscopy show αII-spectrin at node/paranodes, βII-spectrin at paranodes, and βIV-spectrin at nodes in myelinated axons. **(H,J,L)** Images captured by STORM super-resolution microscopy show a periodic lattice of αII, βII, and βIV-spectrins with spacing around 190 nm. **(A–L)** are adapted from Huang et al. (2017a, b). **(M)** Graphic illustration of the periodic spatial organization of the spectrin-based cytoskeleton and its associated proteins in axons. At axonal excitable domains including axon initial segments and nodes of Ranvier, the key molecules for membrane excitability (AnkG, Nav, Kv) in these regions show a similar periodicity. Additionally, spectrins build an intra-axonal boundary (as indicated by red arrow) to restrict excitable domain localizations. Spectrin periodicity in dendrites, however, is less prominent.

By employing super resolution microscopy both in fixed (Xu et al., 2013; Leterrier et al., 2015) and live cultured neurons and brain slice (Xu et al., 2013), spectrins were found in a periodic arrangement along axon shafts with a 190 nm spacing, which matches the length of each purified spectrin tetramer as previously visualized by EM (Bennett et al., 1982). Actin and its capping protein adducin wrap around the circumference of axons as ring-like structures and connect with spectrins throughout the axon (Xu et al., 2013). This pattern is observed in both unmyelinated and myelinated axons (D’Este et al., 2016, 2017), various neuron types *in vitro* (He et al., 2016), and in species from *C. elegans* to human (He et al., 2016). Within axons, the periodicity of spectrins can also be seen at specialized excitable domains like the axon initial segment (AIS) (Figures 1B,D,F) and NoR (Figures 1H,J,L; Zhong et al., 2014; D’Este et al., 2015; Huang et al., 2017a, 2017b). Interestingly, the key molecules for membrane excitability in these regions show a similar periodicity, such as voltage-gated sodium channels (Xu et al., 2013; D’Este et al., 2017), KCNQ2 potassium channels (D’Este et al., 2017), ankyrinG (Leterrier et al., 2015), and the cell adhesion molecule neurofascin (D’Este et al., 2015; Figure 1M). These results suggest the actin-spectrin-based cytoskeleton organizes the subcellular distribution of functional units in axons. It will be interesting to determine if these spatial features participate directly in action potential properties, perhaps by modulating AIS length or position (Grubb and Burrone, 2010; Kuba et al., 2010; Arancibia-Cárcamo et al., 2017).

In contrast to axons, the periodicity of the spectrin-based cytoskeleton is less pronounced in somatodendritic domains (He et al., 2016; Han et al., 2017) and glia (D’Este et al., 2016; He et al., 2016). Spectrin’s periodic pattern in axons is also distinct from the hexagonal network previously described in erythrocytes by EM (Byers and Branton, 1985) and STORM super-resolution microscopy (Pan et al., 2018). These remarkable differences raise several important questions including how is the architecture of spectrin-based cytoskeleton established, what are the key associated molecules involved in this process, how do these mechanisms work in a cell-type or compartment-specific manners, and what is the functional consequence of different spectrin architectures.

## Roles for Spectrins in Axon Integrity

Spectrins allow erythrocytes to deform and withstand the shear forces experienced during vascular flow or as these cells move through capillaries that may be even smaller than the diameter of the erythrocytes themselves. In neurons, axons also encounter distinct types of mechanical stresses including tensile and torque forces due to the movement joints and limbs. The spectrin-based cytoskeleton also helps to maintain axonal integrity under these mechanical forces.

In *C. elegans*, UNC-70 β_G_-spectrin is highly expressed in the nervous system including the nerve rings, nerve cord and commissural axons (Moorthy et al., 2000). Although loss of β_G_-spectrin does not affect neurite outgrowth or integrity during embryogenesis (Hammarlund et al., 2007), after hatching, axons are progressively broken with increasing age, and these axons have aberrant morphology (Hammarlund et al., 2000, 2007; Moorthy et al., 2000). This phenotype can be mitigated by paralyzing the worms (Hammarlund et al., 2007), indicating that spectrins stabilize axons against tensile forces resulting from locomotion. Furthermore, worms lacking β_G_-spectrin cytoskeleton simultaneously with microtubule protein MEC-7 β-tubulin or microtubule-associated protein PTL-1 tau showed large coils and kinks in their axons (Krieg et al., 2017), suggesting these cytoskeletal components are essential to withstand tension and torque forces. In sensory neurons, which experience forces due to touch and movement, the pre-stressed β_G_-spectrin is also required for mechanosensation (Krieg et al., 2014).

In mice, disrupting axonal β-spectrins including βII or βIV-spectrin showed no or only minor axon degeneration (Yang et al., 2004; Zhang et al., 2013). It is possible that loss of these spectrins can be partially compensated as other β-spectrins substitute for and replace those that are lost (Ho et al., 2014). Therefore, to determine the role of axonal spectrins, Huang et al. (2017a) abolished αII-spectrin, the only α-subunit in the nervous system, so that the entire spectrin cytoskeleton is compromised due to the loss of all α/β-spectrin heterotetramers. Mice lacking αII-spectrin in the central nervous system showed widespread axon degeneration (Huang et al., 2017a). Interestingly, loss of αII-spectrin from peripheral sensory neurons also caused axon degeneration, but mostly in large diameter myelinated axons; large diameter axons also show aberrant innervation of proprioceptor and mechanoreceptor nerve endings, while small diameter axons (mainly non-myelinated axons) remained intact (Huang et al., 2017b). The difference between central and peripheral sensory neurons may result from the contribution of the AIS to axon integrity: αII-spectrin is highly clustered at the AIS, a region crucial for maintenance of neuronal polarity in CNS neurons (Huang et al., 2017a), but pseudo-unipolar sensory neurons do not have AIS (Gumy et al., 2017). The vulnerability of large diameter axons to degeneration may also reflect disruption of NoR in αII-spectrin deficient nerves. Huang et al. (2017b) reported that nodes of large diameter axons have more αII-spectrin than small diameter non-myelinated axons. Together these observations suggest that spectrins located at axonal excitable domains may be important for axon survival and integrity. Future studies that abolish spectrin cytoskeletons specifically at the AIS or nodes may help test these ideas.

## Roles for Spectrin at Axonal Excitable Domains

### Axon Initial Segments

Neuronal action potentials are generated at a specialized axonal region proximal to the soma called the axon initial segment (AIS). These regions are 20–40 μm in length and consist of highly concentrated voltage-gated sodium (Nav) and potassium (Kv) channels, which allow highly localized and transient changes in membrane properties to initiate action potentials (Figure 1M). AIS cytoskeletal proteins, especially AnkyrinG (AnkG) and βIV-spectrin, play critical roles in AIS formation and maintenance (Zhang and Bennett, 1998; Komada and Soriano, 2002; Lacas-Gervais et al., 2004; Yang et al., 2007; Hedstrom et al., 2008; Galiano et al., 2012).

How do AIS form and what causes AnkG and βIV-spectrin to become enriched at these sites? Studies in cortical and hippocampal neurons showed that newly differentiated neurons, where axon polarity is defined but AIS are not yet present, αII/βII-spectrin form a complex with AnkyrinB (AnkB) at the distal tip of the axon. As neurons mature, the AnkB/αII/βII-spectrin submembranous cytoskeleton progressively backfills the axon. As this occurs, AnkG, αII/βIV-spectrin begin to be expressed and are targeted to the axon. These cytoskeletal proteins are unable to occupy the same domains as AnkB and αII/βII-spectrin and thus are effectively excluded from the distal axon. Instead, the only place they can form a submembranous cytoskeleton is at the proximal axon where AnkB and αII/βII-spectrin are not located. Thus, this distal cytoskeleton functions like an intra-axonal boundary to restrict AnkG and αII/βIV-spectrin to the proximal axon (Galiano et al., 2012; Figure 1M). Loss of αII- or βII-spectrin disrupted AIS integrity (Galiano et al., 2012, 2017a; Wang et al., 2018b), and manipulating the formation of AnkB/αII/βII-spectrin complexes by gain-of-function approaches both *in vitro* and *in vivo* further determined the role of the complexes as intra-axonal barriers that restrict the length and location of AIS (Galiano et al., 2012). Another study in motor neurons showed that during early development, AnkG is ubiquitously expressed throughout the axon along with AnkB/αII/βII-spectrin. Later on, AnkG is more enriched at the proximal axon, whereas AnkB/αII/βII-spectrin is relatively restricted at the distal domain (Le Bras et al., 2014). The differences of AIS assembly between hippocampal neurons and motor neurons could result from the distinct temporal expression of these key components, myelination, and unique protein interacting partners that occur in a context-dependent manner.

After the AIS is established, βIV-spectrin functions as the major β-spectrin subunit enriched in this domain (Figures 1C,D). Six βIV-spectrin splice variants (βIVΣ1-Σ6) have been identified (Berghs et al., 2000; Tse et al., 2001; Komada and Soriano, 2002), although βIVΣ1 and βIVΣ6 are the two major isoforms expressed in neurons (Komada and Soriano, 2002; Lacas-Gervais et al., 2004; Uemoto et al., 2007). βIVΣ1 is a 280 kDa full-length isoform, whereas the 140 kDa βIVΣ6 is much shorter and with a start site midway through spectrin repeat 10. The temporal expression of these two isoforms is different, as βIVΣ1 is found at developing AIS, while βIVΣ6 expression increases and it becomes the dominant isoform after AIS are established (Berghs et al., 2000; Yoshimura et al., 2016). This observation suggests the AIS spectrin tetramers may be assembled from two different βIV-spectrin isoforms with splice variant switching occurring between early and late developmental stages. Intriguingly, although these spectrins are different lengths, the apparent spacing of actin remains unchanged (Leterrier et al., 2015; Yoshimura et al., 2016). Furthermore, since βIVΣ6 spectrin lacks the CH domain, a non-canonical actin binding motif must exist within βIVΣ6, or there may be other proteins that mediate interactions between spectrin and the actin cytoskeleton.

The roles of βIV-spectrin at the AIS have begun to be determined using a series of transgenic and spontaneous βIV-spectrin mutant mice. βIV-spectrin null mice generated using a gene-trap approach still form AIS in neurons, but the immunoreactivity of Nav and AnkG are dramatically decreased, suggesting that βIV-spectrin is dispensable for AIS formation, but is required to stabilize AIS structure (Komada and Soriano, 2002). This result was also shown in mice lacking only βIVΣ1 (Lacas-Gervais et al., 2004; Uemoto et al., 2007) and in βIV-spectrin mutant mice (*qv^3J^* mice have a frameshift mutation in the C-terminal SD domain of both βIVΣ1 and Σ6) (Yang et al., 2007). These studies confirmed the necessity of the βIVΣ1 variant and βIV spectrin’s C-terminus for AIS integrity. All βIV-spectrin transgenic and mutant mice have tremors, ataxia, and auditory defects, which likely result from the abnormal action potential firing due to AIS disruption.

In addition to its excitable properties, the AIS also functions as a filter to regulate the differential trafficking of somatodendritic and axonal molecules. Previous studies revealed that AnkG-deficient axons lack an AIS, and that in the absence of an AIS axons acquire dendritic features including the entry of dendritic proteins (e.g., MAP2) into axons (Hedstrom et al., 2008).

AnkG and βIV-spectrin may directly function as a diffusion barrier or intracellular “filter” regulating the differential localization of membrane proteins, organelles, vesicles, and even lipids (Leterrier and Dargent, 2014). How do ankyrins and spectrins regulate these functions? One possibility is that spectrins may participate in assembly of a stable platform or scaffold that in turn regulates dynamic actin patches thought to regulate the entry of vesicles and other proteins into the axon (Watanabe et al., 2012; Balasanyan et al., 2017). Although previous studies showed the periodic spacing of spectrins is similar between proximal (βIV-spectrin) and distal axon (βII-spectrin) spectrin cytoskeletons (Xu et al., 2013), whether dynamic changes in cytoskeleton structure occur during protein trafficking through the AIS remains to be determined. We speculate that many other factors including as yet unidentified AIS proteins that interact with βIV-spectrin or AnkG, post-translational modifications, and the splicing variants of βIV-spectrin also contribute to how AIS maintain neuronal polarity.

Despite the very stable nature of the AIS and the common βIV-spectrin and AnkG-based AIS cytoskeleton, AIS morphology, protein composition, and even location can differ among the various types of neurons (Kole and Stuart, 2012; Höfflin et al., 2017); additionally, these features may change in response to external stimuli. Dynamic changes in AIS structure can alter neuronal excitability (Grubb and Burrone, 2010; Kuba et al., 2010). Several mechanisms, including increased intracellular calcium, altering activity and the surface expression of membrane receptors and ion channels, and axo-axonic synapse interaction have been proposed to mediate AIS plasticity (as reviewed in Yamada and Kuba, 2016; Jamann et al., 2018). Nevertheless, the process whereby the underlying cytoskeleton is reorganized remains unknown. Future studies to analyze spectrins using real-time imaging to capture these dynamic changes, super-resolution microscopy, and comparison among different neuron subtypes, may help to uncover how spectrins play roles in AIS heterogeneity and plasticity.

### Nodes of Ranvier

After an action potential is initiated at the AIS, the membrane depolarization propagates along the axon to downstream nerve endings to connect to the next cell in the circuit. In myelinated axons, voltage-gated ion channels are highly enriched at NoR to facilitate saltatory action potential conduction. Nodes are short gaps in the myelin sheath where the action potential is regenerated through the clustered ion channels. Multiple neuron-glia interactions are responsible for the assembly and maintenance of nodes, and impairment of these processes has been linked to disorders including multiple sclerosis (Craner et al., 2004; Howell et al., 2006), Charcot–Marie–Tooth (CMT) disease (Devaux and Scherer, 2005; Saporta et al., 2009), Guillain-Barré syndrome (Yuki and Hartung, 2012), and spinal cord and traumatic brain injuries (Reeves et al., 2010). Nodal spectrin-based cytoskeletons are critical in both health and disease.

Nodes of Ranvier can be divided into three distinct domains: (1) node, where Nav channels are clustered, (2) paranodes, which flank nodes and are the sites where each successive layer of the myelin sheath attaches to the axon, and (3) juxtaparanodes, which are highly enriched in Kv1 K+ channels, immediately adjacent to the paranode, and covered by the myelin sheath. In zebrafish, αII-spectrin is found throughout axons, but can also be found enriched at nodes and paranodes during early development (Figures 1G,H). Mutant zebrafish with an αII-spectrin nonsense mutation at spectrin repeat 13 had diffuse Nav channel intensity, a longer node length, and disrupted paranodal structure, indicating αII-spectrin is required for the proper formation of NoR (Voas et al., 2007). Consistent with this interpretation, mice lacking αII-spectrin in peripheral sensory neurons had ataxia and impaired action potential conduction, disrupted NoR, and axon degeneration (Huang et al., 2017b). Together, these results confirm that αII-spectrin is necessary for node of Ranvier formation across species. However, determining the function of αII-spectrin specifically at nodes or paranodes is challenging due to its presence in both domains. Therefore, studies depleting domain-specific spectrins to disassemble αII/β-spectrin complexes is necessary to understand spectrin’s location-specific functions.

Like at the AIS, nodal βIV-spectrin interacts with AnkG and actin to form a submembranous cytoskeleton (Figures 1K,L). Furthermore, this spectrin cytoskeleton includes both βIVΣ1 and βIVΣ6 (Berghs et al., 2000; Komada and Soriano, 2002; Lacas-Gervais et al., 2004; Uemoto et al., 2007). Mice lacking βIVΣ1 alone had weaker Nav channel intensity at nodes, along with wider and swollen nodal ultrastructure (Lacas-Gervais et al., 2004; Uemoto et al., 2007); these phenotypes were more prominent in βIV-spectrin null mice (Komada and Soriano, 2002; Uemoto et al., 2007) and in the *qv^3J^* βIV-spectrin mutant mice (Yang et al., 2004), confirming the necessity of both isoforms to maintain nodal integrity. Remarkably, restoring the expression of βIV-spectrin in adult βIV-knockout mice mitigates nodal disruption, although the timing is critical (Saifetiarova et al., 2018). Intriguingly, despite weaker Nav channel intensity, compound action potential conduction velocities are intact in βIV-spectrin deficient mice (Yang et al., 2004), suggesting the ataxic phenotypes and early lethality observed in many βIV-spectrin mutant mice may reflect impaired AIS function rather than nodal dysfunction. Consistent with this idea, the percentage of nodes with Nav channels in βIV spectrin mutant mice is not different from controls (Susuki et al., 2013; Ho et al., 2014), suggesting that βIV-spectrin is dispensable for Nav channel clustering, or there are compensatory mechanisms that help to clustering and stabilize nodal Nav channels. For example, βI-spectrin, the major β-spectrin in erythrocytes, is enriched at nodes in the *qv^3J^*βIV spectrin mutant mice (Ho et al., 2014). Interestingly, the expression of βI-spectrin in the dorsal roots of *qv^3J^* mice was comparable to the wild type, indicating there is a pre-existing pool of βI-spectrin in the axons that is not normally found at nodes. Subsequent studies using AnkG knockouts revealed that whether βI or βIV spectrin is found at nodes depends on the type of Ankyrin found at nodes. Although AnkG is the usual nodal Ankyrin, removing AnkG by conditional knockout can be rescued by AnkyrinR (AnkR). Thus, AnkR preferentially interacts with βI spectrin and can rescue Nav channel clustering in the absence of AnkG (Ho et al., 2014). If βI spectrin is normally found in axons but is not located at nodes, what is it doing in axons? Furthermore, since βI-spectrin can compensate for loss of βIV-spectrin to rescue node function, the importance of a nodal spectrin cytoskeleton remains unknown. Future studies that eliminate both βI and βIV-spectrin from the nodes will help to answer this question.

In contrast to βIV-spectrin’s nodal localization, βII-spectrin is found throughout the axon and is enriched at paranodes where it interacts with protein 4.1B (Ogawa et al., 2006; Zhang et al., 2013; Figures 1I,J). Mice lacking βII-spectrin in peripheral sensory neurons have motor dysfunction due to disrupted proprioception, longer nodal length, and juxtaparanodal Kv1 K${}^{+}$ channels that are mislocalized into paranodes (Zhang et al., 2013). However, the axoglial junction itself was unaffected. These results showed that paranodal axonal spectrins are the molecular basis of diffusion barriers flanking NoR (Figure 1M). This function is reminiscent of the intra-axonal boundary that restricts AnkG to the AIS. However, in contrast to AIS (Galiano et al., 2012), loss of the axonal paranodal spectrins did not disrupt the clustering of nodal AnkG or Nav channels (Zhang et al., 2013). This difference from AIS indicates that although some mechanisms are shared between the two, additional clustering mechanisms exist at nodes. At AIS an intra-axonal cytoskeletal barrier clusters AnkG and βIV spectrin, while at NoR two glia-dependent mechanisms recruit AnkG and converge on axonal spectrins (Susuki et al., 2013; Amor et al., 2017).

βII-spectrin is also found in Schwann cells at paranodes, and mice lacking βII-spectrin in myelinating glial cells have disrupted paranodal axoglial junctions in young mice. These defects eventually resolve, but then re-appear as mice age, suggesting that glial βII-spectrin is required for the timely formation and long-term maintenance of paranodal junctions (Susuki et al., 2018).

## Spectrins in Neurological Disease and Injury

### Pathogenic Spectrin Variants

Heterozygous mutations in *SPTAN1* lead to early infantile epileptic encephalopathy-5 (EIEE5, OMIM# 613477) (Table 1), which is characterized by seizures with hypsarrhythmia, intellectual disability and delayed development. Several mutations have been identified near spectrin repeat 20-21 (Saitsu et al., 2010; Hamdan et al., 2012), which mediates α/β-spectrin dimerization. Biochemical analyses showed that complexes formed between mutant αII-spectrin and βII or βIII-spectrins are less thermostable. Furthermore, the mutant αII-spectrin also causes spectrin aggregation, disruption of AIS structure including reduced Nav channel clustering, and impaired action potential firing (Saitsu et al., 2010; Hamdan et al., 2012). Mice expressing human pathogenic *SPTAN1* variants have shortened or no dendrites and a smaller cell soma. Neurons induced from patient-derived iPSC also have less complex neuronal processes and spectrin aggregations (Wang et al., 2018b). These phenotypes were highly similar to *SPTAN1* null animals (Huang et al., 2017a; Wang et al., 2018b), indicating that pathogenic *SPTAN1* variants may behave in a dominant negative manner.

**TABLE 1:** Pathogenic spectrin variants and associated neuropathies.

**Gene symbol**	**Protein**	**Pathological genotypes**	**Associated disease**	**Clinical features**	**Histological features**	**References**
*SPTAN1*	αII-spectrin	In-frame deletion, Duplication	EIEE5 (OMIM# 613477)	Early onset epileptic encephalopathy, Lack of visual attention, Intellectual disability	Brain atrophy, Thin corpus callosum, Hypomyelination	Saitsu et al., 2010; Hamdan et al., 2012; Writzl et al., 2012; Tohyama et al.,2015
*SPTBN2*	βIII-spectrin	In-frame deletion, Missense mutation, Frame-shift mutation, Missense mutation	SCA5 (OMIM# 600224), SPARCA1 (OMIM# 615386)	Motor incoordination	Cerebellar atrophy, EAAT4 accumulation in Purkinje cell	Ikeda et al., 2006; Lise et al., 2012; Parolin Schnekenberg et al.,2015
*SPTBN4*	βIV-spectrin	Nonsense mutation, Missense mutation, Frameshift	βIV-spectrinopathy (OMIM#606214)	Congenital hypotonia, Intellectual disability	Muscle fiber atrophy, Weaker Nav and KCNQ2 channel intensity at nodes	Knierim et al., 2017; Wang et al.,2018a

Mutations in *SPTBN2* give rise to spinocerebellar ataxia type 5 (SCA5, OMIM# 600224) (Table 1), which is characterized by adult-onset and progressive motor incoordination, postural abnormalities and swallowing difficulties. Autopsy tissue from heterozygotic patients with an in-frame deletion at spectrin repeat 3 showed significant loss of Purkinje cells and a thinner molecular layer in the cerebellum. This mutant βIII-spectrin allele also impaired the synaptosomal localization of the glutamate transporter EAAT4 in Purkinje neurons, which may be an underlying cause of the neurodegeneration (Ikeda et al., 2006). Another nonsense recessive allele in *SPTBN2* caused developmental cerebellar ataxia and cognitive impairments during childhood. These phenotypes are distinct from SCA5, and this developmental disorder is called spectrin-associated autosomal recessive cerebellar ataxia type 1 (SPARCA1) (Lise et al., 2012; Table 1). Nevertheless, how these pathogenic variants give rise to different neurological signatures remains poorly understood. Additional studies are required to reveal the functions, expression profiles and associated proteins of βIII-spectrin in the nervous system; these studies may reveal how these different *SPTBN2* variants give rise to distinct disorders.

Several different pathogenic variants in *SPTBN4* were recently described (Knierim et al., 2017; Wang et al., 2018a). These patients all have a remarkably similar disease including congenital hypotonia, developmental delay and intellectual disability from early childhood; some patients also have seizures and central deafness (OMIM# 606214) (Wang et al., 2018a; Table 1). Wang et al. (2018a) investigated the consequences of these mutations and found that for many, their capacity to interact with AnkG and to be clustered at the AIS was compromised. For other mutations near the C-terminus, the mutant βIV-spectrin protein was unable to bind to phosphoinositides. These properties are essential to stabilize the cytoskeletal architecture of the AIS and to cluster ion channels at AIS and NoR. Future studies to determine the impact of spectrin pathogenic variants on other AIS functions, such as regulating polarity through control of intracellular trafficking, may help determine the cause of pathological phenotypes in these patients.

### Proteolysis of Spectrins After Injury

Spectrin breakdown products (SBP) have been widely used as biomarkers for central nervous system injury for three main reasons. First, the Ca^2+^-dependent cysteine protease calpain is activated after CNS injury and cleaves full length αII-spectrin (280 kDa) into 150 and 145 kDa fragments (Pike et al., 2001). These SBP can be isolated from cerebrospinal fluid, allowing clinical evaluation. Second, the quantitative and temporal expression of SBP are highly correlated with the severity of injury and affected regions as evidenced by multiple injury models in rodents, which renders SBP as useful indicators to evaluate the degree of trauma. Finally and most importantly, the breakdown of spectrin-based cytoskeleton faithfully reflects axonal damage, as shown by the disruption of AIS (Schafer et al., 2009) and axon degeneration (Yang et al., 2013). These strengths render SBP as powerful indicators of axon injury and degeneration.

How is the cleavage of αII-spectrin regulated? There are at least two known mechanisms. First, Src-kinase phosphorylates αII-spectrin at Tyr1176 (adjacent to the SH3 domain and cleavage site) which reduces the susceptibility to proteolysis by calpain (Nicolas et al., 2002; Nedrelow et al., 2003). Second, binding between calpain and its endogenous inhibitor calpastatin maintains homeostatic enzymatic activity. However, after injury, increased intracellular [Ca^2+^] tips the balance toward activated calpain which degrades the calpastatin. This exacerbates calpain-mediated proteolysis of αII-spectrin (Yang et al., 2013). Transgenic mice that constitutively overexpress human calpastatin (hCAST) in neurons have reduced levels of αII-spectrin SBP and improved motor performance after injury (Schoch et al., 2012). Exogenous delivery of calpastatin into injured neurons also mitigates axon degeneration (Yang et al., 2013). Many calpain inhibitors have now been developed for therapeutic purposes, although improvements in efficacy and safety are still needed [for review see (Ono et al., 2016)]. It is also important to emphasize that αII-spectrin is but one of calpain’s many targets and functional deficits in neurons are not due solely to disruption of the spectrin cytoskeleton.

Increased levels of SBP have been reported in many neurological disorders other than CNS injury, including amyotrophic lateral sclerosis (Yamashita et al., 2012), Guillain-Barré syndrome (McGonigal et al., 2010), Parkinson’s disease (Diepenbroek et al., 2014) and acute inflammatory autoimmune demyelinating models (Guyton et al., 2010; Das et al., 2013). Inhibition of calpain activity in these studies generally reduced SBP levels. Future studies that examine not only the integrity of axons, but also the structures whose function depends on specialized spectrin-based cytoskeletons (e.g., AIS and NoR), may help to define the pathophysiology of these diseases and injuries. Additionally, it will be important to determine whether the protective effects of calpain inhibitors is due to their protection of glial or axonal spectrins.

## Conclusion

Since their original description in erythrocytes, our understanding of spectrins has dramatically increased and these important cytoskeletal proteins are recognized as being essential to the functions of diverse cell types in the nervous system. With the advent of advanced microscopy techniques and genetically modified mice, the roles of spectrins are now becoming much clearer. This knowledge will not only help to define the basic function of the nervous system, but it may also suggest therapeutic strategies for nervous system disorders and injuries that include altered or disrupted spectrin cytoskeletons.

## Author Contributions

Both authors wrote the manuscript.

## Conflict of Interest Statement

The authors declare that the research was conducted in the absence of any commercial or financial relationships that could be construed as a potential conflict of interest.
